# Contextual cuing survives an interruption from an endogenous cue for attention

**DOI:** 10.3758/s13414-024-02966-8

**Published:** 2024-10-10

**Authors:** Tom Beesley, Louise Earl, Hope Butler, Inez Sharp, Ieva Jaceviciute, David Luque

**Affiliations:** 1 Department of Psychology, Lancaster University, UK, Lancaster, LA1 4YD UK; 2 Universidad de Málaga, Málaga, Spain

**Keywords:** Visual search, Incidental learning, Contextual cuing, Attention, Endogenous cuing

## Abstract

Three experiments explored how the repetition of a visual search display guides search during contextual cuing under conditions in which the search process is interrupted by an instructional (endogenous) cue for attention. In Experiment 1, participants readily learned about repeated configurations of visual search, before being presented with an endogenous cue for attention towards the target on every trial. Participants used this cue to improve search times, but the repeated contexts continued to guide attention. Experiment 2 demonstrated that the presence of the endogenous cue did not impede the acquisition of contextual cuing. Experiment 3 confirmed the hypothesis that the contextual cuing effect relies largely on localized distractor contexts, following the guidance of attention. Together, the experiments point towards an interplay between two drivers of attention: after the initial guidance of attention, memory representations of the context continue to guide attention towards the target. This suggests that the early part of visual search is inconsequential for the development and maintenance of the contextual cuing effect, and that memory representations are flexibly deployed when the search procedure is dramatically interrupted.

It is well established that the process of visual search is guided by past experience. When we encounter a scene, the extent to which the present stimuli match representations in memory will determine the effectiveness of the stimulus processing and subsequent search through the scene. The contextual cuing (CC) task is a common way to study this cognitive process in the lab: participants typically experience a standard visual search task (i.e., serial processing; slow search), such as searching for a T amongst L shapes. A set of search configurations is repeated across trials, and response times to targets are faster compared to those in configurations that do not repeat. Thus, the repetition of the search configurations leads to the formation of a representation of the configuration in memory, and future processing of the same configuration activates this representation, driving more efficient behavior within that scene.

Much work has focused on the nature of the memory and attention processes responsible for contextual cuing. The effect was initially suggested to be implicit in nature, with repeated configurations seemingly guiding search unconsciously: typically participants are unable to articulate their knowledge of the repeated configurations, and show poor ability to recognize learned configurations in memory tests (e.g., Chun and Jiang, 1998; Colagiuri and Livesey, 2016), although this view of CC has been strongly contested (e.g., Smyth & Shanks, 2008; Vadillo et al., 2016). There are also a number of plausible computational models of how memory representations of repeated configurations are formed and result in the CC effect (e.g., Beesley et al. 2015; Brady and Chun, 2007). The predominant view is that the memory representations are best characterized as associative in nature, whereby distractors (or groups of distractors, see Beesley et al. (2016)) form associations that activate more strongly the contingent target position within each repeated configuration.

The exact nature of how repeated configurations come to facilitate visual search is the focus of much debate within the literature. Broadly, there are two quite distinct theoretical accounts of why responses are faster for repeated configurations: the early attentional guidance account, and the late response facilitation account. According to the early account, recognition of the configuration leads to a more efficient search process through the distractor array, such that the target is localized (fixated) at an earlier time point in search. Perhaps the clearest (and arguably simplest) evidence in support of this account comes from studies of eye-tracking during CC. For example, search through repeated configurations results in fewer fixations prior to target localization (e.g., Beesley et al., 2018; Tseng and Li, 2004). According to the late response facilitation account, the benefit for repeated configurations comes about as a result of enhanced target processing once it has been localized by attention. One conceptualization of this process is that repeated configurations lead to a reduction in the evidence threshold required to ascertain that the target is present in its location, such that responses can be initiated earlier. Such an account has been put forward by Sewell et al. (2018), in order to explain the evidence supporting the late account from response time modeling of the CC effect.

It seems likely that both early and late processes contribute to the overall CC effect (for a review see Sisk et al.  (2019)). The current article focuses on exploration of the early-stage attentional account of CC. The term “early” here reflects the fact that the CC benefit is present prior to the detection of the target and the initiation of the response to the target. In fact, the “early” phase can be further divided. Analysis of eye movements has shown that serial visual search can be defined as having two distinct phases: an initial ineffective search in which the direction of saccades is inconsistent, and a secondary effective phase in which each saccade will draw attention closer to the target. CC appears to result from having more trials with a shorter ineffective phase.

One interpretation of these data is that CC is initially not driven at all by the configuration, and that the initial distractor processing is not beneficial for the representations that form for repeated configurations. Supporting evidence for this account comes from Olson and Chun (2002), where participants were trained on a CC task in which either all the distractors repeated, those in the half of the screen containing the target (short-range-context), or those in the half of the screen that did not contain the target (long-range context). CC was observed in the short-range context, but not in the long-range-context condition. Thus, it would appear that the distractors further from the target are not critical to the generation of a CC effect.

Brady and Chun (2007)’s computational account features a mechanism that ensures spatial constraints are placed on the learning of associations with relation to their proximity to the target. If the spatial constraints are tuned to modulate learning and restrict associative formations to only those distractors close to the target, this model can accurately model the data from Olson and Chun (2002). Since the only consequential mechanism in the model for CC is the associative weights (and their modulation by spatial constraints), then one prediction that follows from this account is that the initial phase of search is inconsequential for observing CC.

In contrast to the localized facilitation account, it is possible that contextual cuing involves learning of a procedural template that guides eye movements in a consistent manner following experience with the task. A recent study by Seitz et al. (2023) has provided evidence to support this claim. Participants’ eye movements were monitored for repeated and randomly arranged configurations, and similarity metrics were computed to identify the consistency of scan-paths over time. Several findings point to the establishment of a more general type of procedural learning in CC. Firstly, it was found that scan-path similarity increased over the course of training, but that the similarity of scan-paths was higher in repeated compared to random configurations. Secondly, scan-path similarity was higher in the initial half of the search trial compared to the second half. These data suggest that, in contrast to the earlier characterizations of the initial search process as “inefficient”, this early phase may be an important part of the behavioral response in CC, potentially involving the development of a generic scanning behavior.

Importantly, Seitz et al. (2023) suggest that CC is best characterized as involving the acquisition of this generic procedural scanning response, and a configuration-specific facilitation. These behaviors occur in the early and late period of oculomotor guidance, respectively. The question remains as to how critical the early activation of procedural knowledge is to the development of CC. The current article examines this by significantly interrupting the search process with an endogenous cue for attention. In all experiments, participants complete a contextual cuing visual search task but are also presented with an arrow that signals the side of the screen on which the target will appear. Thus, this cue disrupts the natural search process, considerably reducing the operation of the generic scanning response in the early phase of search. The experiments therefore examine whether this initial part of the search process is inconsequential for the observation of the CC effect, or whether the development and maintenance of the generic scanning behavior contributes substantially to the CC effect.

In the current experiments, we explored how CC is affected by the interruption of the search process by a clear direction of attention from an endogenous cue. In Experiment 1, we examine whether a learned pattern of behavior is disrupted due to the onset of the endogenous cue, while in Experiment 2 we seek to establish whether the CC effect is weaker under these conditions. Experiment 3 explores the underlying drivers of the CC effect, in terms of the distractor-target associations, during these procedures.

## Experiment 1

Experiment 1 sought to examine whether the learned attentional behavior that develops during contextual cuing is still expressed when participants are directed by an endogenous (instructional) cue to search in a particular region of the visual scene, hindering the operation of early stage visual search processes. Participants were first trained with a set of four repeating configurations in phase 1 across 5 epochs of 32 trials each. Then, prior to phase 2, participants were told that an arrow would appear before every trial indicating the side of the screen on which the target would be located. This arrow was valid on every trial. In phase 2, the repeating configurations were presented in two forms: “consistent”, where the target appeared in the same position as it has appeared for that configuration in phase 1; and “inconsistent”, where the target appeared in a position in the opposite quadrant of the screen from where it had appeared in phase 1. Random configurations were also presented in this phase. The inclusion of the inconsistent trials in this phase provides a test of whether the distractors processed in the early stages of search continue to guide attention in the presence of the endogenous cue. If this is the case, we would also expect that the contextual cues would guide attention *away* from the (new) target quadrant on inconsistent trials, and so response times should be slower on these trials compared to those on random trials.

### Method

#### Participants

Thirty-one undergraduate students from Lancaster University were recruited (mean age = 20.1, SD = 1.1; 17 identified as female and 14 as male) via the Psychology Research Participation System in the Department of Psychology at Lancaster University, in return for the opportunity to use the recruitment system for their own research in future years. Analysis of the current experiments was performed with Bayesian methods, seeking support for either the null or alternative hypothesis on critical tests. As such, we aimed for the maximum sample size that could be achieved by the resources of the experimenter (approximately 30). These sample sizes were similar to much of our previous lab work with contextual cuing tasks.

#### Materials

Participants were tested individually in a quiet room with a Dell laptop with a 15.6” screen, a screen resolution of 1920 x 1080, and a full-size external keyboard for participants to use to respond to the task. Participants sat approximately 50 cm from the screen. Stimulus presentation was controlled by MATLAB using the Psychophysics Toolbox extensions (Brainard, 1997; Kleiner, Brainard & Pelli, 2007; Pelli, 1997). Responses to the target stimulus were made by pressing the ‘c’ or ‘n’ key on a standard keyboard. All experimental materials are available at the http://github.com/tombeesley/CC_EC repository for this study.

Distractor stimuli were an ‘L’ shape (rotated 0$^{\circ }$, 90$^{\circ }$, 180$^{\circ }$, or 270$^{\circ }$) while the target stimulus was a ‘T’ shape (rotated at either 90$^{\circ }$ or 270$^{\circ }$). Stimuli were 8 mm square and arranged in a square grid of 144 evenly spaced cells (12 x 12) which was positioned centrally on the screen and was 170 mm square. The grid itself was invisible to participants. The fixation cross (displayed centrally before each trial) was 4 mm square. The background of the screen was grey (RGB: .6, .6, .6) and the stimuli were presented in black (RGB: 1, 1, 1). There was a small offset in the vertical line of the ‘L’ distractors, which increased the similarity between the ‘L’ distractors and the target ‘T’, making the search task more difficult (Duncan & Humphreys, 1989).

#### Design

Phase 1 employed a within-subjects design with factors of epoch (1–5) and configuration (repeated and random). All configurations contained 16 distractors, equally divided between the four quadrants of the display, and one target. Four repeated configurations were trained^1^. Four target locations were used, with one from each quadrant assigned to each of the repeated configurations. These same four target positions were used for the random configurations throughout the task. Each of these four target positions was chosen at random from one of five locations within each quadrant, which were approximately equidistant from the center of the screen. Distractors could not appear in these target locations.

**Fig. 1:**
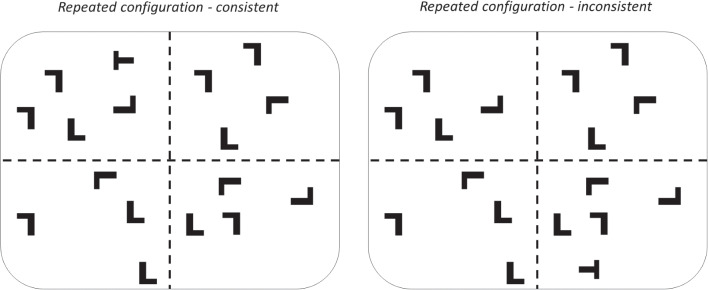
Schematic of the manipulation of target position in consistent and inconsistent trials of phase 2. The *dashed lines* show the division of the stimuli into quadrants, but were not present in the task procedure

Phase 2 employed a within-subjects design with factors of epoch (6–10) and configuration (repeated: consistent; repeated: inconsistent; random: consistent; random:inconsistent). On each trial, there was a .5 probability that an “inconsistent” version of the configuration would be presented. This meant that the target was relocated to a diametrically opposed target position such as to maximize the displacement from the trained target position (see Fig. 1). This could occur for both the repeated and random configurations, hence creating four unique trial types for this phase. While random configurations did not have a “trained”, associated, target position, it is necessary to divide the random trials into consistent and inconsistent trial types in this way in order to assess any target frequency effects that may occur, since the inconsistent target locations used in this phase were novel.

#### Procedure

Participants were tested individually in a quiet testing room. They were given instructions on how to complete the task, including the presentation of an example of a search trial. Participants were shown the two correct responses for the two possible orientations of targets.

Each trial commenced with a fixation cross presented in the center of the screen for 500 ms, which was then replaced immediately by the search configuration. Participants searched for the target stimulus and responded with a left or right response depending on its orientation. Reaction times (RTs) were recorded from the onset of the search configuration. Following a valid response (c or n), the configuration was removed from the screen. The ITI was 1000 ms. If participants made an incorrect response to the target orientation, the text “INCORRECT RESPONSE” appeared in red in the center of the screen for 3000 ms, prior to the ITI. If participants did not respond within 6000 ms, “TIMEOUT - TOO SLOW” appeared in red in the center of the screen for 3000 ms, prior to the ITI.

Each block of eight trials contained each of the four different repeated configurations and four random configurations. These eight configurations could appear in any order with the constraint that the position of the target did not repeat across trials or across consecutive blocks.

A rest break of 30 s was given every 80 trials. Trials started automatically after these breaks. After 160 trials, prior to phase 2, participants were given an instruction screen which detailed the arrow that would appear on the screen prior to the configuration. They were able to ask any questions they had at this stage and then proceeded to phase 2. The arrow appeared for 1000ms following the fixation cross, before the presentation of the search configuration. The task was otherwise identical to that used in phase 1.

### Results

Our criterion for removing outlier data, at both the participant level and the trial level, was 2.5 standard deviations above or below the mean of the sample. On average, trials ended with a timeout on 1.97% of trials (SD = 2.53). Two participants had an usually high proportion of timeouts and were removed from the analysis. The mean accuracy of participants (not including timeout trials) was 98.10% (SD = 1.65%). One participant had an unusually low proportion of accurate trials and was also removed. The only participant deemed to be an outlier in terms of mean response time (hereafter RT) was also excluded on the basis of the timeout criterion, noted above. For the remaining 28 participants we removed trials with a timeout and inaccurate trials, before removing outliers from the RT data. On average, the proportion of outliers removed was 3.03% (SD = 0.79%). Zero participants had an unusual proportion of trials removed as outlier RTs (greater than 2.5 SDs above the mean).

**Fig. 2:**
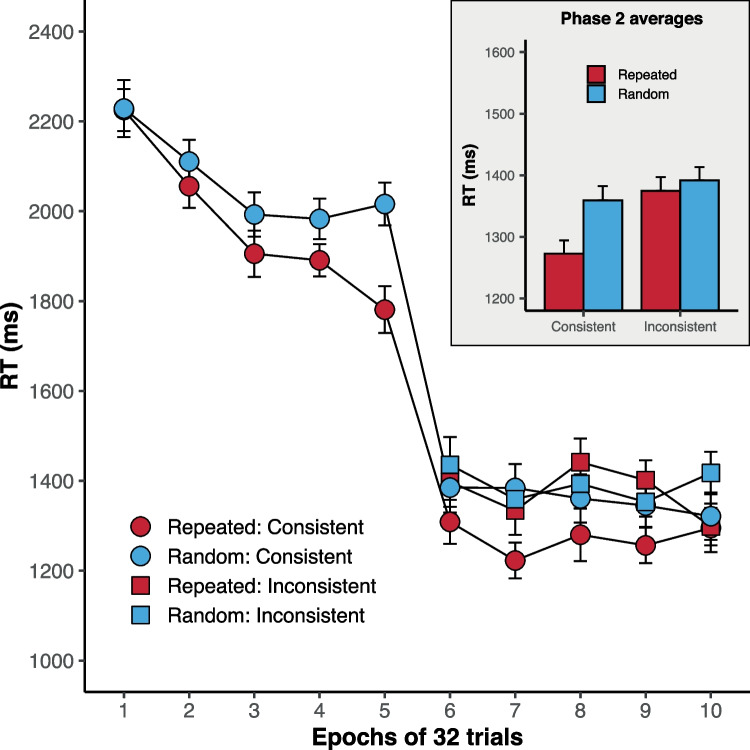
RT data for Experiment 1. The phase 2 averages across the four trial types are shown inset. Within-subject *error bars* were computed by a process of normalizing the RT data for the sample (Cousineau, 2005)

Figure 2 shows the RT data across the ten epochs of the experiment. In phase 1 (epochs 1–5) a contextual cuing effect emerged, with faster responses to repeated over random configurations. In phase 2, the presence of the guiding arrow led to a clear reduction in the response times. For all participants, the mean RT across epochs 4 and 5 was higher than the mean RTs across epochs 6 and 7. Despite the clear evidence for the processing of the endogenous cue, the underlying search configuration continued to play a role in the guidance of attention, with faster response times for (consistent) repeated configurations compared to random configurations.

These data were analyzed with a Bayesian ANOVA^2^, using the *BayesFactor::anovaBF()* function in R (version 4.4.0; (R Core Team, 2024)). All analyses in this study used the default parameters for the priors, which “places mass in reasonable ranges [of effect sizes] without being overcommitted to any one point” (Rouder et al., 2017, p. 317). First taking the data from phase 1 (epochs 1–5), there was strong support for the model containing the factors of epoch and configuration (repeated vs. random), BF_10_ = 2.2 $\times$ 10^12^ ± 3.88%. The addition of the interaction term did not improve the model fit, BF = 0.44 ± 3.95%, though there was no evidence for the absence of the interaction. The best-fitting model was a better fit than the two models containing only one of the factors, smallest BF = 36.57 ± 3.89%, providing strong support for both the effects of configuration and epoch. Partial eta-squared ($n^2_p$) effect sizes were calculated using *effectsize::eta_squared*, giving values of: 0.22 for the effect of configuration; 0.39 for the effect of epoch; and 0.1 for the interaction effect.

A Bayesian ANOVA on the data from phase 2 (epochs 6–10) found strong support for the model containing the factors of configuration (repeated vs. random) and target position (consistent vs. inconsistent), BF_10_ = 46.61 ± 1.08%. The next best-fitting model contained these two factors and the interaction term, and was not a substantially worse fit to the data, BF = 0.56 ± 1.82%. The best-fitting model (with factors of configuration and target position, but no interaction) was a substantially better fit to the data than the model containing only the factor of configuration BF = 20.7 ± 1.36% providing evidence that RTs were faster on consistent than inconsistent trials. There was no evidence for a difference between the best-fitting model and the model containing only the factor of target position, BF = 2.36 ± 1.48%. The relevant effect sizes ($n^2_p$) were: 0.14 for the effect of configuration; 0.22 for the effect of target position; and 0.14 for the interaction of these two factors.

To further explore responses to the different trial types in phase 2, Bayesian *t*-tests were run using *BayesFactor::ttestBF* (using the default Cauchy prior) for comparisons between the repeated and random configurations, across the two target position conditions (consistent and inconsistent). This revealed substantial support for a difference between the response times on “repeated: consistent” trials and those on the respective random trials (random: consistent), BF_10_ = 4.14 ± 0%. There was also substantial evidence to suggest there was no meaningful difference between the response times for the “repeated: inconsistent” trials and the respective random trials, BF_10_ = 0.24 ± 0.03%.

To compare the size of the CC effect across phases 1 and 2, we calculated a “CC effect score” by subtracting the RT on consistent repeated trials from the RT on consistent random trials. Positive values reflect a CC effect. There was a CC effect score of 142.72 ms (SD = 202.68) for the end of phase 1 (epochs 3–5) and a CC effect score of 106.76 ms (SD = 176.12) for the start of phase 2 (epochs 6–8). A Bayesian *t* test of the effect of phase on CC effect found moderate support for the null result, BF = 0.25, suggesting that the CC effect was not attenuated in the second phase.

### Discussion

In Experiment 1, we established a contextual cuing effect in the first phase, before introducing an endogenous cue for attention that directed the participants consistently towards the side of the screen on which the target was presented. Unsurprisingly, this had a dramatic effect on reducing RTs in all participants, but there remained a significant contextual cuing effect in this second phase. Thus, disrupting a substantial part of the early search process did not appear to affect the performance of the contextual cuing that had been established: notably there was evidence to suggest that the contextual cuing effect in phase 2 was of a similar magnitude to that which was observed in phase 1. On some repeated trials in phase 2, we positioned the target in a diametrically opposed location on the screen. On these trials, there was no impact of the repeated configuration on performance.

These findings together support a view of contextual cuing in which the initial process of search is inefficient, not being guided in any way by the repeated context. Only when attention lands within a region of space approaching the target does the repeated configuration take over to guide search efficiently towards the target. It should be acknowledged that the variable search behavior that participants would exhibit during the early part of the search process would naturally lead them to search the area around the target on many trials. As such, on trials without the endogenous cue, the termination of the inefficient phase of search will occur earlier on some trials compared to others. The cuing of attention by the valid arrow cue ensures this termination happens on every cued trial, eliminating the inefficient phase of search.

The maintenance of a robust contextual cuing effect in phase 2, in the presence of the endogenous cue, suggests that CC is a robust and flexible behavior. The apparent ability to entirely disrupt and negate the early part of the search process, while maintaining an intact CC effect, is at odds with the “general procedural learning” that has been suggested to occur in CC (Seitz et al., 2023). According to this account, “...what may look like an ineffective phase [of search] actually constitutes an important period during which procedural learning of a general scanning scheme becomes functional.” (Seitz et al., 2023, p. 9). The present data suggest that this aspect of search can be eliminated at no cost to CC.

## Experiment 2

In Experiment 1, we demonstrated that an established effect of contextual cuing is maintained even when attention is being guided by the presence of a valid endogenous cue. That is, we found that the *performance* of an established search behavior in contextual cuing is not disrupted by the guidance of attention. In Experiment 2, we wanted to explore whether the *learning* of the contextual cue itself was affected by the presence of a valid endogenous cue. That is, does the presence of a valid endogenous cue, which leads to a controlled command of attention, limit the development of a contextual cuing effect. To do this, we trained each participant on two sets of repeating configurations. One of these sets was always presented in the presence of a valid endogenous cue, while the other set was always presented in the absence of the endogenous cue. The extent to which there is a “cue-competition” effect between the endogenous cue and the contextual cues can be examined by comparing the contextual cuing effect we observe for the two sets of configurations. Given the clear difference in RTs we observed in Experiment 1 between the trials with the endogenous cue present and the cue being absent, we anticipated the same difference in responding in Experiment 2. Therefore, we also included a second phase of Experiment 2 in which we removed the endogenous cue entirely from the task. This second phase therefore allowed us to directly compare the contextual cuing for the two sets of configurations when RTs were at a comparable level.

Given the results of Experiment 1, we would anticipate that the size of the CC effect would be comparable in the two conditions. That is, Experiment 1 suggests that the CC effect is unaffected by the presence of the endogenous cue, and therefore that the effect is reliant on the cuing that occurs by distractors later in the search process. Removal of the inefficient period of search should not dramatically affect the development of CC.

### Method

#### Participants

Thirty-four undergraduate students from Lancaster University were recruited (mean age = 20.74, SD = 5.29; 28 identified as female and six as male) via the Psychology Research Participation System in the Department of Psychology at Lancaster University, in return for the opportunity to use the recruitment system for their own research in future years.

#### Materials

Participants were tested in a quiet laboratory testing cubicle, with a standard PC and a 24” monitor set at a resolution of 1920 x 1080 pixels. Since the monitor was larger for this experiment, the dimensions of the presented stimuli had a proportional increase in size: Distractor stimuli were 11 mm square; the search grid was 240 mm square; the fixation cross was 6 mm square. In all other respects, the materials were the same as those detailed in Experiment 1.

#### Design

Four repeated configurations were created in an identical manner to those used in Experiment 1. For each participant, two of these configurations were used for the condition in which the arrow cue was presented before the configuration, while two were used for the “control” condition (no arrow presented). As in Experiment 1, the four repeated configurations were paired with unique target positions from each of the four quadrants. We counterbalanced the use of the target quadrants across the factors of configuration type (repeated and random) and cue condition (arrow vs. no-arrow). For half of the participants, targets in the top left and bottom right were used for the repeated configurations presented with the arrow (cue-competition) condition, with targets in the top right and bottom left used for repeated configurations in the no-arrow (control) condition. For these participants, random configurations presented with the arrow had targets in the top right and bottom left, and random configurations without the arrow had targets in the top left and bottom right. For the other half of the participants, these assignments were reversed (repeated-arrow: top-right and bottom-left; repeated-no arrow: top-left and bottom-right; random-arrow: top-left and bottom-right; random-no arrow: top-right and bottom-left).

#### Procedure

The procedure was the same as Experiment 1 with the following differences. Participants received 320 trials in total. For the first 160 trials, the arrow was presented for the relevant conditions. For the final 160 trials, the arrow was never presented. Rest breaks were given every 60 trials.

### Results

Our criteria for removing outlier data were identical to Experiment 1. On average, trials ended with a timeout on 2.13% of trials (SD = 1.83). Zero participants had an unusually high proportion of timeouts. The mean accuracy of participants (not including timeout trials) was 95.85% (SD = 6.10%). One participant had an unusually low proportion of accurate trials and were removed from the sample. Zero participants were deemed to be an outlier in terms of mean RT.

For the remaining 33 participants, we removed trials with a timeout and inaccurate trials, before removing outliers from the RT data. On average, the proportion of outliers removed was 2.81% (SD = 1.04%). One participant had an unusual proportion of trials removed as outlier RTs and was not included in the final analysis.

**Fig. 3:**
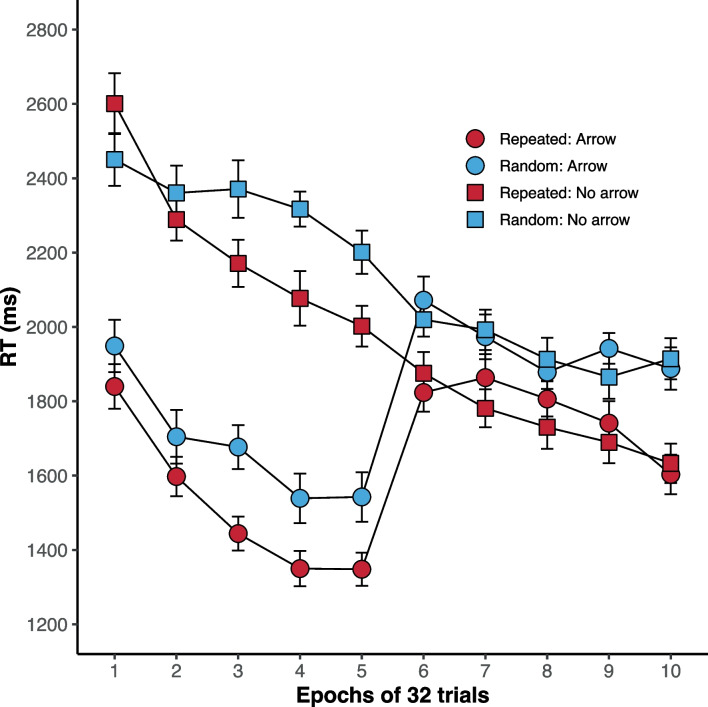
RT data for Experiment 2. *Error bars* show standard error of the mean on normalized data

Figure 3 shows the RT data across the ten epochs of the experiment. Contextual cuing emerged rapidly in both the arrow and no-arrow conditions, with little suggestion that the CC effect was different in the two conditions. The phase 1 data were explored with a Bayesian ANOVA, which revealed that the best-fitting model contained the factors of epoch, configuration (repeated vs. random), and endogenous cue (arrow present vs. arrow absent), with no interaction terms, BF_10_ = 7.5 $\times$ 10^100^ ± 3.98%. The next best-fitting model contained all three factors and the interaction of epoch and configuration, BF_10_ = 5.2 $\times$ 10^100^ ± 1.28%, and this model was not a substantially worse fit to the data, BF = 0.7 ± 4.18%. All other models were substantially worse fits than the best-fitting model, largest BF = 0.26 ± 1.66%. Importantly, the interaction term between the factors of endogenous cue and configuration did not improve the fit of the model, providing substantial support for the absence of this interaction, BF = 0.18 ± 4.12%. The relevant effect sizes ($n^2_p$) were: 0.44 for the effect of epoch; 0.4 for the effect of configuration; 0.85 for the effect of endogenous cue; 0.12 for the interaction effect between configuration and epoch; and 0.02 for the interaction between configuration and endogenous cue.

When the endogenous cue was removed in the second half of the experiment, RTs were equivalent across the two conditions. An effect of configuration was seen for both cuing conditions, with little discernible difference between the size of the cuing effects. We conducted a Bayesian ANOVA on the data from the second phase, with factors of epoch, configuration, and endogenous cue condition (whether the configuration had been trained in the context of the arrow or no-arrow in phase 1). The best-fitting model was that with just the factors of epoch and configuration with no interaction between the factors, BF_10_ = 9.6 $\times$ 10^14^ ± 1.37%. There was substantial support for this model over the next best-fitting model, BF = 8.94 ± 2.06%. To examine the interaction of the configuration and endogenous cue factors, we compared the model containing those two factors to the model containing the two factors plus the interaction of configuration and endogenous cue, which revealed substantial support for the absence of an interaction, BF = 0.13 ± 3.04%. The relevant effect sizes ($n^2_p$) were: 0.62 for the effect of configuration; and 0.25 for the effect of epoch.

To provide further support for the absence of the interaction between the factors of configuration type and endogenous cue, the data from across the experiment (epochs 1–10) were analyzed with a Bayesian ANOVA with only the factors of configuration and endogenous cue. The best-fitting model was that with the two factors and no interaction, BF_10_ = 3.7 $\times$ 10^51^ ± 1.95%. The addition of the interaction term did not strengthen the model, with substantial evidence for the absence of the interaction, BF = 0.09 ± 2.42%. The relevant effect sizes ($n^2_p$) were: 0.77 for the effect of the endogenous cue; and 0.61 for the effect of configuration.

### Discussion

Experiment 2 sought to examine whether the presence of a valid endogenous cue would impair the acquisition of a contextual cuing effect. In the first phase, two sets of configurations were trained, one of which was always presented in the presence of the endogenous cue, and one set which was presented without the endogenous cue. Overall, there was considerable evidence that the cue was processed and acted upon, as response times to the target were much faster on cued trials. However, there was no evidence to suggest that the instructed guidance of attention impaired the acquisition of the configurations on those trials. Furthermore, when the endogenous cue was never presented in the final phase of the experiment, the size of the contextual cuing effect was equivalent between the two sets of configurations; the Bayesian analyses found support for the equivalence of these CC effects.

The data from Experiment 2 are consistent with the findings of Experiment 1: the early phase of search is inconsequential for the development of contextual cuing. The equivalence of the CC effects across the two groups (cued and uncued) would suggest that the guidance by the context was driven entirely by the distractors that appear close to the target. The longer search times in the uncued condition clearly indicate that a far greater number of distractors are processed in this condition, but that the enhancement of attentional guidance by the repeated distractors is limited to the later part of the search process, and therefore those nearer to the target. Alternatively, it is at least possible that the repeated distractors are processed rapidly at the onset of the trial, before the effects of the endogenous cue on attention are observed. If this is the case, then those repeated distractors that influence search (producing the CC effect) need not be localized around the target. Experiment 3 provides a test of these two possible accounts.

## Experiment 3

As noted earlier, the analysis of eye movements during contextual cuing tasks (Beesley et al., 2018; Tseng & Li, 2004) has revealed a characteristic scanning pattern comprising two phases: search initially occurs in an inefficient manner, as the eyes move between distractors in the central region of the distractor field, before then moving in a more directed manner towards the target position. Contextual cuing appears to result from a cessation of the inefficient search phase at an earlier time point in the entire search process, such that processing of repeated distractors will, on average, result in fewer fixations. With respect to the current study, in Experiments 1 and 2 we have initially directed attention towards the side of the screen that contains the target on cued trials. This will bring about an early cessation of the first phase of the search process. From here, however, it seems that search is still facilitated by the repetition of the context.

To test this characterization of the interaction between the endogenous cue and the repeated context, we exposed participants to the same procedure as used in phase 1 of Experiment 1, which establishes a contextual cuing effect prior to the use of the endogenous cue. In a second phase, we then presented the endogenous cue on every trial (as in Experiment 1), but we manipulated the presence of the repeated distractors within the configurations. For each repeated configuration we created two variations: in the “proximal” configurations, only the distractors in the quadrant containing the target match those from the full repeated configuration, while the distractors in the other three quadrants were randomly arranged on each trial; in the “distal” configurations, the distractors closest to the target were randomized, while the distractors in the other three quadrants were the same as those in the full repeated configuration. During this phase, we also presented fully repeated configurations and fully randomized configurations. Comparison of the response times across these four trial types will allow us to determine the contribution of proximal and distal distractors to the CC effect when attention is cued endogenously.

### Method

#### Participants

Forty-two undergraduate students from Lancaster University were recruited (mean age = 18.64, SD = 2.84; 28 identified as female and 14 as male) via the Psychology Research Participation System in the Department of Psychology at Lancaster University, in return for the opportunity to use the recruitment system for their own research in future years.

#### Materials

All materials, including stimuli and testing environment were identical to Experiment 2.

#### Design

The design of phase 1 was identical to Experiment 1, with four repeated configurations created and presented with random configurations during this phase. For phase 2, each of the four configurations was manipulated to create two alternative conditions. In the “Repeated distal” condition, the four distractors in the target quadrant were randomly arranged on each trial, while the 12 distractors in the other three quadrants were presented in the same positions as had been trained in phase 1. Thus, slower response times for this condition (compared to the fully repeated configurations) would indicate the extent to which participants CC was governed by the distractors closest to the target. For the “Repeated proximal” condition, the four distractors in the target quadrant were presented in the same positions as had been trained in phase 1, while the 12 distractors in the other three quadrants were randomly arranged on each trial. Thus, slower response times for this condition (compared to the fully repeated configurations) would indicate the extent to which CC was governed by the distractors further from the target. Comparison of the RTs for these different configurations with those of the random configurations would allow for the assessment of whether these subsets of distractors had *any* contribution to the CC effect that had developed during phase 1.

#### Procedure

The procedure was identical to Experiment 1.

### Results

Our criteria for removing outlier data were identical to Experiment 1. On average, trials ended with a timeout on 2.81% (SD = 2.25) of trials. Two participants had an usually high proportion of timeouts and were removed from the sample. The mean accuracy of participants (not including timeout trials) was 96.09% (SD = 8.57%). Two participants that had an unusually low proportion of accurate trials were also removed. Zero participants were deemed to be an outlier in terms of mean RT.

For the remaining 38 participants, we removed trials with a timeout and inaccurate trials, before removing outliers from the RT data. On average, the proportion of outliers removed was 3.17% (SD = 0.71%). Zero participants had an unusual proportion of trials removed as outlier RTs.

**Fig. 4:**
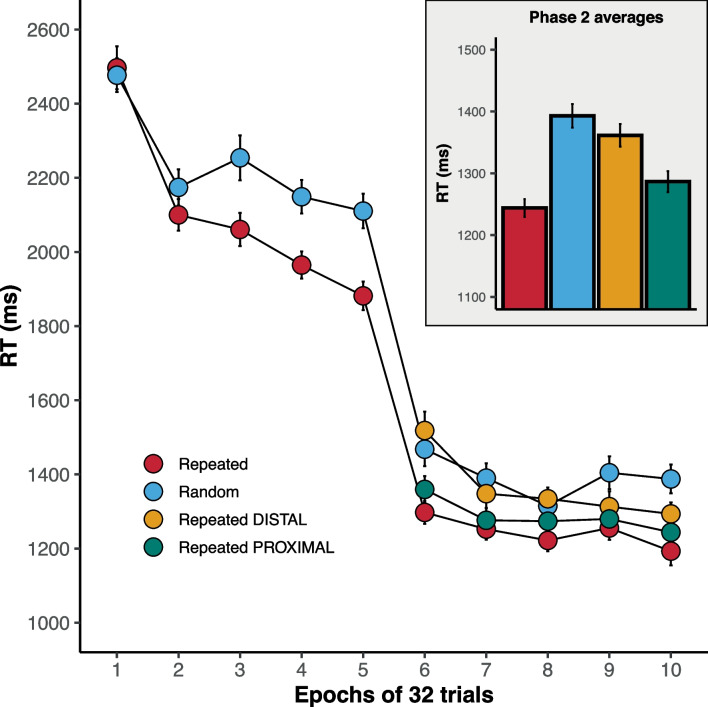
RT data for Experiment 3. *Error bars* show standard error of the mean on normalized data

Figure 4 (main panel) shows the RT data across the ten epochs of Experiment 3. As in Experiment 1, contextual cuing was readily established in phase 1. These data were subjected to a Bayesian ANOVA, which revealed that the best-fitting model contained the factors of configuration (repeated vs. random) and epoch, and an interaction between those factors, BF_10_ = 5.3 $\times$ 10^24^ ± 0.96%. However, the model without the interaction provided a strong fit to the data, BF_10_ = 5.1 $\times$ 10^24^ ± 0.61%, and a comparison between the two models did not find any evidence in support of the interaction term, BF = 0.96 ± 1.14%. There was strong support for the best-fitting model over the remaining models, smallest BF = 3881.73 ± 1.15%, providing strong support for the factors of epoch and configuration. The relevant effect sizes ($n^2_p$) were: 0.38 for the effect of the epoch; and 0.47 for the effect of configuration; and 0.08 for the interaction of these two factors.

The response times decreased significantly with the presentation of the valid endogenous cue in phase 2. Response times to the fully repeated configurations were somewhat comparable to those when just the proximal repeated distractors were present. Response times for the distal repeated distractors appeared to be slower and comparable to the fully random configurations. The phase 2 data were subjected to a Bayesian ANOVA, which found that the best-fitting model contained the factors of configuration and epoch but no interaction between the factors, BF_10_ = 1.4 $\times$ 10^14^ ± 0.64%. This model provided a superior fit to the data compared to the next best-fitting model that included the two factors and the interaction term, BF = 123.65 ± 1.03%, providing strong support for the contribution of the two factors and the absence of an interaction between the two factors. The relevant effect sizes ($n^2_p$) were: 0.37 for the effect of configuration; and 0.16 for the effect of epoch.

The inset graph in Fig. 4 shows the mean RTs to the four types of configuration, averaged across the five epochs of phase 2. To explore the differences in response times, Bayesian *t*-tests were run for all pairwise comparisons. The response times to repeated and repeated-proximal configurations were both faster than those to random configurations, smallest BF_10_ = 10313.81 ± 0%. In contrast, there was no evidence that the response times to repeated-distal configurations were different from those to random configurations, BF_10_ = 0.39 ± 0.04%. Response times to repeated configurations were faster than those to repeated-proximal configurations, BF_10_ = 4.67 ± 0%. Response times to repeated-proximal configurations were faster than those to repeated-distal configurations, BF_10_ = 31.88 ± 0%.

### Discussion

Experiment 3 explored the localization of the distractors driving contextual cuing when attention is guided by an endogenous cue. As expected, there was substantial evidence that contextual cuing was present when the distractors close to the target were maintained, but not when these distractors were randomly arranged. These data appear to confirm a clear order to the interplay between the two drivers of attention: initially attention is guided by the endogenous cue towards one half of the screen, and then search is refined by the presence of the valid configural cues (the repeated distractors). Like in Experiment 1, the phase 2 data demonstrate the resilience of the CC effect to changes in the search process. Despite visual search never commencing in a cued manner during the initial acquisition period of phase 1, a CC effect was readily observed in phase 2. Thus, it seems that the stored representations of configurations surrounding target positions are flexibly deployed in visual search, despite changing demands on controlled attentional processes. Notably, the fully repeated configurations exerted more of a benefit on search than those containing only the proximal distractors, suggesting that the repeating distractors beyond the target quadrant have some (but possibly lesser) influence on search (Brady & Chun, 2007).

These data lend support to the notion that the effect of the repeated configuration is a late process within visual search, and that each trial commences with an inefficient search process that is not guided by the repeated configuration (Beesley et al., 2018; Tseng & Li, 2004). In some ways, these findings represent a paradox of CC: the cuing effect occurs almost at the point at which target detection has been made. One interpretation would be that this demonstrates the importance of spatial contiguity in the formation of visual associations (Renaux et al., 2017). Alternatively, it provides support for the proposed “decision threshold” accounts of CC (Kunar et al. 2007; Sewell et al., 2018), which posit that the repeated distractors close to the target ensure a reduced threshold for target detection, resulting in faster response times.

## General discussion

Three experiments explored the impact of a central endogenous cue of attention on the contextual cuing of visual search. In Experiment 1, having established a contextual cuing effect, each trial was preceded by a central endogenous cue of attention in the form of an arrow, directing attention towards the side of the screen in which the target was positioned (this arrow cue was always valid, as was the case in each of the three experiments). Despite participants clearly using this cue, visual search was still facilitated by the presence of the repeating pattern of visual search. This experiment demonstrated that, once acquired, the activation of the memory representation and its impact on performance of visual search remains intact in the presence of a top-down instruction to guide attention. Experiment 2 examined the storage of these contextual representations, and whether this process was impaired by an endogenous cue guiding search. We found equivalent levels of contextual cuing for configurations trained with the endogenous cue and those trained in its absence. Together, these two experiments suggest a seamless interplay between these two factors governing attention in visual search: the endogenous cue initially guides attention and the repeated configuration continues to refine and guide attention towards a fixation on the target. In Experiment 3 we therefore explored whether the localized distractors around the target were sufficient to generate CC following the guidance by the endogenous cue. Indeed, there was a significant CC effect in the case of the proximal distractors, but repeated configurations that did not contain the proximal distractors failed to generate a CC effect, suggesting that the proximal distractors play a crucial role in search following the guidance of attention by the endogenous cue.

Our data are consistent with previous theoretical (Brady & Chun, 2007) and empirical (Olson & Chun, 2002) work that has highlighted the influence of distractor configurations localized to the target. Experiment 2 in particular demonstrates that acquisition of effective representations is equivalent if search is limited to one-half of the display from the outset. In Experiment 3 the CC effect was observed only when fully repeated and proximal-repeated configurations were presented. Interestingly the CC effect was substantially weaker in the case of configurations with only proximal-repeated distractors. This must reflect a generalization decrement between the stored representation and the available cues for the target. Our manipulation of the influence of repeated distractors was based on disrupting the repeating configurations on a quadrant basis: those inside the quadrant retained their positions, while those outside were randomized. This somewhat crude manipulation will not perfectly capture the impact of all distractors: it is likely that the influence of distractors at increasing distances from the target will have a gradually reducing influence on driving a CC effect.

The current data reveal that the influence of repeated contexts has a relatively late control on behavior in visual search. Previous analysis of eye-movements during CC (Beesley et al., 2018; Tseng & Li, 2004) has shown that contextual cuing (and visual search more generally) has two characteristic components. The first of these is an inefficient search process where search fails to move towards the target in trials with more fixations. This is followed by a phase in which monotonic, positive increments are made toward the target position in the final 3 to 4 fixations. CC reduces the frequency of trials with the initial search period (there are more of such trials for random configurations and fewer for repeated configurations). Search behavior under CC conditions is necessarily variable, however, and each time a configuration is encountered, the pattern of eye-movements will inevitably be driven by a range of factors that lead to variation in the scan path taken. What is clear is that it is the final few fixations and saccades that are crucial to the search behavior that facilitates CC, and this period will follow a variable length of ineffective search. Thus, the effect of the endogenous central cue in the current study is to eliminate, or considerably reduce, the engagement with this first phase of the search process. The results of this study strongly imply that the positive associative information in the repeating configurations is extracted in the final stages of search and is localized around the target. This is true both in terms of the performance of an acquired configuration (Experiments 1 and 3) and the acquisition of the representation for that configuration (Experiment 2).

Recently, work by Seitz et al. (2023) has suggested that CC is made up of both configuration-specific learning, and eye-movements that reflect “...procedural learning of a general scanning scheme...” [p. 9]. This latter aspect of the acquired CC behavior was suggested to occur within the earlier period of inefficient search. If such a behavior developed in our CC task, it is clear that this behavior is not critical to the performance of the CC effect (in Exp 1 and Exp 3), or to the development of the learned behavior that drives the CC effect (Exp 2). Contrary to the suggestion by Seitz et al. (2023), it is at least possible that such general procedural learning has some influence over the later stages of search. However, such an account would have to assume that this learnt behavior is flexible enough to survive the curtailment of a considerable portion of the pattern of eye-movements. We would argue it is simpler to account for the present data by assuming the expression of a pattern-specific sequence of eye-movements that occurs late on in the search process, following the period of ineffective search.

The current data are also consistent with a late-stage “response threshold” account of CC (Sewell et al., 2018). According to this perspective, the facilitation for repeated configurations occurs because the target is more readily detected amongst the surrounding distractors. Analysis of ERPs has revealed enhanced contra-lateral delay activity (CDA) for repeated over random configurations. This is thought to reflect “postselective (focal-attentional) processing of items held in working memory” (Chen et al., 2022). In the present tasks, such a mechanism would not be affected by the onset of the endogenous cue and the curtailing of the period of ineffective search. Taken together, the results here point towards the possibility of three components to the behavior in CC: an early ineffective search, followed by enhanced localization and increased perceptual discrimination of the target, driven by the distractors closest to the target.

The effect of CC on visual search has frequently been characterized as an automatic influence on behavior (e.g., Chun and Jiang, 1998; Chun and Nakayama, 2000; Geyer et al. 2021). This characterization of CC comes from multiple aspects of the observed effect. Updating of the associations is somewhat slow and seemingly inflexible to changes in the acquired associations (Makovski & Jiang, 2011; Manginelli & Pollmann, 2009; e.g., Zellin et al., 2013), and therefore perhaps reflects a habitual form of behavior. In addition, contextual cuing has frequently been observed in the absence of above-chance recognition memory for the repeating search configurations (e.g., Colagiuri and Livesey, 2016), which suggests a non-conscious, automatically evoked form of behavior. Despite this persistent characterization, the automaticity (or controllability) of CC has rarely been directly tested in the literature. To our knowledge, only the experiments of Luque and colleagues (Luque et al., 2017; Luque et al., 2021) have directly assessed this aspect of CC, by placing the influence of the configuration in competition with top-down goals in the task. Their findings supported the conclusion that CC performance can be controlled and will not guide search for the target when another aspect of the task governs attentional control. In the current study, the repeated configurations continued to have an influence on search performance even when attention had been guided by the endogenous cue. In this respect, it might be suggested that these results are somewhat at odds with the conclusions of Luque and colleagues (Luque et al., 2017; Luque et al., 2021).

To what extent is this behavior best characterized as “automatic” in nature? Arguably the clearest demonstration of an automatic effect of a stimulus on behavior is when the associated behavior is elicited even when it is counter-productive to the current goals (Moors & De Houwer, 2006). We could argue that such a test was constructed in the repeated inconsistent trials of Experiment 1, in which the repeated configuration was associated with a target that was previously located in a position on the opposite side of the screen to the direction indicated by the endogenous cue. If the repeated configuration had an effect on behavior on these trials, we would have expected to see slower response times compared to random trials. This was not the case: response times were equivalent in these two conditions. As such it is hard to claim here that the configuration is having an *automatic* effect on behavior, according to this strict characterization of such an effect. Nevertheless, the experiments here reveal a flexible interplay between top-down drivers of attention and configuration-driven effects of attention in CC.

## Data Availability

The raw data and experimental materials are freely available at the project repository http://github.com/tombeesley/CC_EC

## Footnotes

1 Though CC experiments may frequently train eight or 12 repeated configurations, it has been established that participants typically learn only a subset of these (Smyth & Shanks, 2008). In order to avoid simply adding unnecessary noise to our measure of CC, our lab typically trains just four repeated configurations in our CC procedures.

2 The Bayesian analyses here follow the process outlined in Rouder et al. (Rouder et al. (2017)). Briefly, we present the best-fitting model evaluated against the null model, and then compare this fit to that of other models. Where the comparison of two models (i.e., A against B) reveals a Bayes factor of greater than 3, this is taken as support for the components of model A that are not present in model B. Bayes factors of less than 0.33 are taken as evidence in support of the equivalence of two models. Following Wetzels et al. (2011) we use the terms “substantial” (BF>3; BF<1/3), and “strong” (BF>10; BF<1/10) to reflect the levels of support for the results of the model comparisons.

## Code Availability

The data analysis scripts and the manuscript source files are available at http://github.com/tombeesley/CC_EC. The analyses reported in this manuscript are computationally reproducible from the manuscript source files (using R v4.4.0).
